# A multimodal deep learning radiomics model for predicting degenerative meniscus tear after arthroscopy

**DOI:** 10.1371/journal.pone.0328299

**Published:** 2025-08-13

**Authors:** Yao He, Jiaying Wei, Yinsong Sun, Wei Bao, Denghua Huang, Yuanjun Fan, Wei Huang, Tingting Wang

**Affiliations:** 1 Department of Orthopedics, Banan Hospital of Chongqing Medical University, Chongqing, China; 2 Department of Orthopedics, The First Affiliated Hospital of Chongqing Medical University, Chongqing, China; 3 Chongqing Municipal Health Commission Key Laboratory of Musculoskeletal Regeneration and Translational Medicine, Chongqing, China; 4 Orthopedic Research Laboratory of Chongqing Medical University, Chongqing, China; 5 General Committee Office, Banan Hospital of Chongqing Medical University, Chongqing, China; Shanghai Jiaotong University: Shanghai Jiao Tong University, CHINA

## Abstract

**Background:**

Degenerative meniscus tears are often accompanied by varying degrees of osteoarthritis, making the prognostic outcome of arthroscopic partial meniscectomy (APM) difficult to predict. Our research objective is to develop and validate a multimodal deep learning radiology (MDLR) model based on the integration of multimodal data using deep learning radiology (DLR) scores from preoperative magnetic resonance imaging (MRI) images and clinical variables.

**Materials and methods:**

From February 2020 to February 2022, 452 eligible patients with degenerative meniscus tear who underwent APM were retrospectively enrolled in cohorts. DLR features were extracted from MRI of the patient’s knee. Then, an MDLR model was used for the patient prognosis after arthroscopy. The MDLR model for prognostic risk stratification incorporated DLR signatures and clinical variable.

**Results:**

The standalone DLR model performed poorly, with a micro average receiver operating characteristic (ROC) curve and macro average ROC line of 0.780 and 0.765 in the training set, 0.747 and 0.747 in the validation set, and 0.720 and 0.732 in the test set, respectively, for predicting postoperative outcomes in degenerative meniscus tears. Multivariate analysis identified gender, height, weight, duration of pain, ESR, and VAS as indicators of poor prognosis. After combining the above clinical features, the performance of the MDLR model has been significantly improved, with the best performance achieved under the Light Gradient Boosting Machine (GBM) algorithm. The micro average ROC curve and macro average ROC line of this model for predicting the postoperative effect of degenerative meniscus tear were 0.917 and 0.919 in the training set, 0.874 and 0.882 in the validation set, and 0.921 and 0.951 in the test set, respectively. With these variables, the MDLR model provides four levels of prognosis for arthroscopic partial meniscectomy: Poor, pain relief 0–25%, Average, pain relief 25–50%, Good, pain relief 50–75%, Excellent, pain relief 75–100%.

**Conclusion:**

A tool based on MDLR was developed to consider that the pain exacerbation time is an important prognosis factor for arthroscopic partial meniscectomy in degenerative meniscus tear patients. MDLR showed outstanding performance for the prognostic efficiency stratification of degenerative meniscus tear patients who underwent arthroscopic partial meniscectomy and may help physicians with therapeutic decision making and surveillance strategy selection in clinical practice.

## Introduction

Knee joint pain is the most common type of joint pain, accounting for 5% of all outpatient visits [1]. Among the various causes of knee pain, meniscus tears rank among the top three worldwide, along with osteoarthritis (OA), which affects 654 million people and constitutes 23% of adults over age 40, [2] and patellofemoral pain (PFP), which has a lifetime incidence of about 25% [3]. Meniscus tears impact 620 million adults, representing 12% of the general adult population [4].

The meniscus is a fibrocartilaginous structure in the knee joint, made up of two crescent-shaped parts: the medial and lateral menisci. These structures help distribute weight and contribute to joint stability [5]. Meniscal tears, which refer to the separation of this fibrous tissue, can be classified as either traumatic, caused by excessive shear forces, or degenerative, resulting from repeated stress on a worn-out meniscus. According to the report, the annual incidence of clinically diagnosed meniscal tears is 79 cases per 100,000 people (95% CI, 63–94) [6]. Acute traumatic tears are most commonly seen in young, active individuals aged 18–40 who regularly participate in sports and often experience accompanying anterior cruciate ligament injuries [7].

For traumatic meniscal tears, several randomized controlled trials have established more standardized treatment protocols. Depending on the type of tear, either conservative treatment or arthroscopic surgery may be utilized, and the postoperative outcomes are generally satisfactory[8–13].

Degenerative tears typically occur in older adults, particularly those aged 40 and above, and are often associated with knee osteoarthritis. A population-based study in the United States found that 63% of older adults with symptomatic osteoarthritis had meniscal tears detected by MRI [14]. Additionally, meniscal tears are frequently discovered incidentally during MRI examinations. A meta-analysis revealed that 19% (95% CI, 13%−26%) of individuals aged 40 and over, who had no prior history of knee pain or injury, had asymptomatic meniscal tears detected by MRI, [15] and the degenerative meniscal tears are generally non-traumatic and a normal consequence of aging. This condition is often linked with knee osteoarthritis and accompanying degenerative changes [16].

There is no consensus on the best treatment methods for degenerative meniscal tears, various randomized controlled trials (RCTs) have produced differing results [17–20]. One systematic review found that certain factors—such as a prolonged symptom duration (over one year), radiographic evidence of osteoarthritis, and a meniscectomy rate exceeding 50%—were linked to poorer clinical outcomes following partial meniscal resection [21]. Many experts and medical associations recommend partial meniscectomy as a suitable option for treating patients with mild to moderate osteoarthritis or those with meniscal tears who have not benefitted from physical therapy or other non-surgical treatments. They suggest that the prognosis for degenerative meniscal tears largely depends on the associated osteoarthritis [22,23].

One of the main reasons for this different outcome is that we cannot directly assess the nature of osteoarthritis in patients with degenerative meniscus tears using MRI, nor can we conduct a thorough evaluation of the patients’ overall condition. This creates significant uncertainty in predicting the prognosis for patients following arthroscopic surgery.

Despite the high accuracy of MRI features in predicting meniscal tears and osteoarthritis injuries, previous studies have shown that potential selection bias from inter-observer variation is challenging to eliminate. However, deep learning (DL), a data-driven approach, is increasingly being utilized to automatically develop and organize predictive capabilities based on specific features rather than relying on human assessment [24–26].

In recent years, deep learning has become prevalent in medical image analysis, primarily due to its unique advantages in handling multidimensional and large-scale data. Its applications in medical imaging mainly include: image and examination classification; object and lesion classification; organ, region, and landmark localization; target or lesion detection; segmentation of organs and substructures; lesion segmentation; medical image registration; content-based image retrieval; image generation and enhancement; and automatic generation of image reports [27]. Among these, image and examination classification is one of the first areas where deep learning has made significant contributions to medical image analysis. In examination classification, one or more images (an examination) are typically used as input to produce a single diagnostic variable as output (for example, the presence or absence of disease). In this context, each diagnostic examination is a sample, and compared to the dataset sizes in computer vision, the dataset sizes are generally smaller (for example, hundreds or thousands versus millions of samples) [28]. Therefore, the popularity of deep learning in such applications is not surprising.

In the past decade, DL as a data-driven approach, has been increasingly applied towards automatic design and organization based on the predictive ability of specific features instead of human performance [29,30]. An increasing number of deep learning methods have been proposed in orthopedic and sports medicine practices to diagnose meniscus tears and osteoarthritis [24,31]. However, relevant research using deep learning to predict postoperative prognosis models for patients with degenerative meniscus tears undergoing arthroscopy is sparse.

Therefore, further research is necessary to support the accuracy of DLR methods in predicting postoperative prognosis for degenerative meniscus tear patients. The objective of our study is to develop and validate a multimodal DLR model based on preoperative MRI and laboratory tests to predict the prognosis of patients with degenerative meniscus tears undergoing arthroscopic partial meniscectomy, utilizing multimodal data that integrates clinical variables and DLR scoring.

## Materials and methods

This retrospective study protocol received approval from the Institutional Review Board at participating hospital and was conducted in accordance with the principles of the 1975 Helsinki Declaration. Because the study is retrospective, the requirement for written informed consent was waived. The study was approved by the Banan Hospital of Chongqing Medical University review board. And the research study was registered in the Chinese Clinical Trial Registry (registration ID: ChiCTR2500098922).

### Patient enrolment

We conducted data retrieval within the three-month period from October 15, 2024 to January 15, 2025 and 452 cases in total of degenerative meniscus tear were diagnosed during APM performed in our hospital from February 2020 to December 2022. A degenerative tear was defined as a slowly developing lesion occurring or insidious onset without any history of trauma [32,33], and appearing as a horizontal cleavage (intrameniscal linear signal often communicating with the articular surface), radial, or complex tear pattern on MRI, [34] and this was confirmed intraoperatively. The inclusion criteria are: 1) Age ≥ 40 years old. 2) Diagnosed as meniscus tear. 3) During hospitalization, arthroscopic surgery diagnosed degenerative meniscus tear. 4) Partial meniscectomy was performed to treat the meniscus. The exclusion criteria are: 1) Patients diagnosed with traumatic meniscus tear based on their medical history and intraoperative conditions. 2) Accompanied by ligament injuries around the joints, such as anterior and posterior cruciate ligament injuries, medial and lateral collateral ligament injuries, patellar ligament injuries, etc. 3) Merge with other types of arthritis, such as gouty arthritis, Charcot’s disease, tuberculous arthritis, rheumatoid arthritis. 4) The patient was admitted for conservative treatment and did not undergo surgery.

### Data set

**Fig 1:**
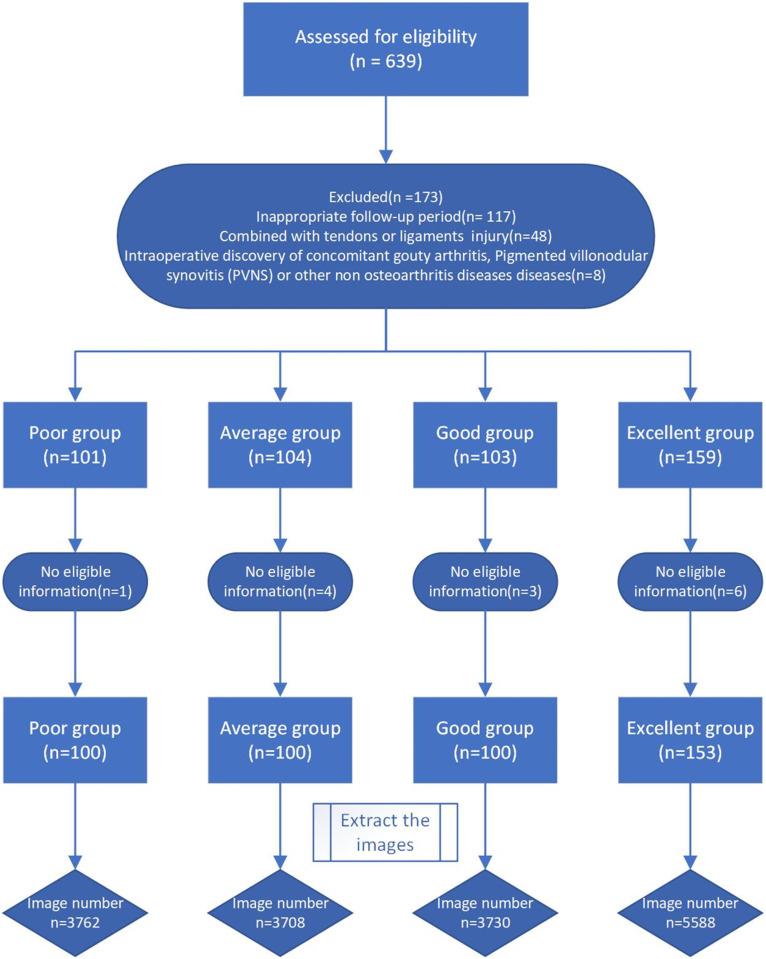
Flowchart of the numbers of patients and images.

Imaging data collected all images of T1 and T2 sequences of the sagittal plane of the knee joint magnetic resonance imaging of patients before operation, and each patient had about 40 images. The clinical characteristics of the included patients were extracted according to the possible influencing factors found in previous studies, including gender, height, weight, body mass index (BMI), white blood cell (WBC), neutrophil percentage, blood glucose, erythrocyte sedimentation rate (ESR), pain time and pain aggravation time, Fig 1 shows the numbers of patients and images included in this study. To protect the confidentiality of patients, patient data were fully anonymized before analysis.

### Surgical Procedure, Rehabilitation and Follow‑up protocol

All arthroscopic procedures were carried out without a tourniquet using Laryngeal Mask Anesthesia. The standard anterolateral and anteromedial portals, along with a 5 mm arthroscope, were employed. We conducted APM in every patient to restore the meniscus’s regular structure. Damaged and loose segments of the meniscus were excised utilizing a mechanical shaver and meniscal basket. The approach aimed to retain as much healthy meniscal tissue as possible. Damaged cartilage was not treated during surgery and no medications were administered into the knee during or after the arthroscopic procedure.

The rehabilitation program consisted of progressive neuromuscular and strength training exercises spread over 12 weeks, occurring twice a week. These exercises were designed to maintain range of motion, improve flexibility in the hips and hamstrings, increase strength in the quadriceps and hips, and preserve knee proprioception.

The primary endpoint for this study was 2 years follow-up, and the primary outcome was patient satisfaction with the current function of their knee. At 2 years after surgery, the patients underwent clinical and radiological evaluations, including answering the following question regarding their perception of the clinical outcomes of APM. Satisfaction was elicited using a 5-point Likert scale (1, very dissatisfied; 2, somewhat dissatisfied; 3, neither satisfied nor dissatisfied; 4, somewhat satisfied; and 5, very satisfied). The results were then Divide the results into four levels, with values of 5 assigned to “excellent”, 4 assigned to “good”, 3 assigned to “average” and values of 1 and 2 assigned to “poor”.

### Study design

Image preprocessing: the image is processed as follows before being input into the deep learning network: 1. resampling to 128 × 128 × 128 by linear interpolation; 2. standardize image blocks with Z-score; 3. enhance images by randomly rotating −30–30 °, without other methods such as panning and zooming. to combat uncertainty in clinical application scenarios.

Deep learning model development: This study designed a hybrid deep learning model architecture MobileNet based Hybrid Network (MobHy-Net), using MobileNetV2 as a powerful feature extraction tool, removing the last layer and classification layer, and only obtaining the feature map we need. Specifically, we segment the features into fixed sized blocks and perform multi head self attention calculations on these different blocks to obtain blocks containing positional information. The processed blocks are classified by a classifier, and the fused information is decoded by a multi-layer perceptron. To avoid overfitting caused by data imbalance, a weighted oversampling method and a binary cross entropy loss function were used. The MobileNetV2 architecture and formula are shown in File S1.

**Fig 2:**
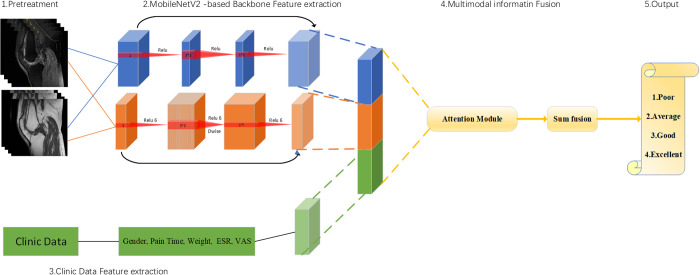
Overall flowchart of the model.

Patients (not individual images) were randomly assigned to training, validation, and test cohorts (7:2:1 ratio) using patient-level stratified splitting to ensure no data leakage across cohorts. The training and validation queues are used to update model parameters and optimize hyperparameters, respectively. The overall flowchart of the model are shown in Fig 2. Using MobHy-Net as the backbone network and pre training the dataset in the DLR model。

### Clinical information integration

Independent sample t-test was conducted on clinical features to remove features with p > 0.05. The Least Absolute Shrinkage and Selection Operator (LASSO) algorithm was used to construct a penalty function λ to shrink some regression coefficients to force some features to become 0, thereby incorporating stable features into LASSO analysis. Based on the maximum average cross validation score standard, perform 3 folds cross validation to determine the optimal lambda value. Select clinical features with non-zero coefficients based on the model corresponding to the optimal λ value. Thus obtaining independent and stable clinical features.

Using neural network to extract combined image features to get DL-sign, and combining the screened clinical features with DL sign and establish a joint model using 14 algorithms including logistic regression, Naive Bayes, Support Vector Machine, Decision Tree, Random Forest, Extra Trees, XGBoost, AdaBoost, Multi-Layer Perceptron, Gradient Boosting Machine, etc. in predicting postoperative outcomes. The above modeling was completed using the PixelmedAI platform.

### Statistical analysis

Statistical analysis was performed using the packages of python software version 3.12.7 (https://www.python.org). The dataset was divided into four groups with postoperative patient satisfaction as the reference standard. The clinical and imaging features logical differences among the four groups were compared using Chi-squared, Kruskal-Wallis and one-way ANOVA. Simultaneously, the training, test and validation cohorts were separated to compare the significant difference using one-way ANOVA. Two-tailed tests were used in all statistics, and variables with P < 0.05 were considered statistically significant. Cross-validation was described in terms of the accuracy of each fold and mean accuracy.

The data set was split into training, validation and test groups (7:2:1). Due to the total sample size in our study being less than 500, in order to ensure sufficient training samples for each fold, we set the K value to 3. The training group was used for K-fold stratified validation to validate the models. Over 1200 images for the each group were used randomly for each fold. The folds were made by preserving the percentage of samples for each class so that the distribution of the data sets did not interfere [35]. After validating and finetuning the model, we evaluated the model on the test set. The test set was split before training and was not used for training or validation.

The model performance of the test set was evaluated using the predictive accuracy (ACC), Positive predictive value (PPV), Negative predictive value (NPV), area under curve (AUC). F1-score, sensitivity, and specificity. The confusion matrix is also described to reflect the sensitivity and specificity of the model, so as to evaluate the performance of the algorithm. The predictive accuracy and F1 score were calculated as follows:


$$Predictive\;accuracy\;=\frac{TP+\;TN}{TP\;+\;FP\;+\;FN\;+\;TN}\mathrm{\backslash ]}$$

$$Positive\;predictive\;value\;(PPV)=\frac{TP}{TP+FP}\mathrm{\backslash ]}$$

$$Negative\;predictive\;value\;(NPV)=\frac{TN}{TN+FN}\mathrm{\backslash ]}$$

$$F1-score=2*\frac{\left(Senstivity\;*\;Positive\;predictive\;value\right)}{\left(Senstivity+\;Positive\;predictive\;value\right)}\mathrm{\backslash ]}$$

where TP indicates a true positive, TN indicates a true negative, FP indicates a false positive, and FN indicates a false negative.

## Results

Table 1 lists the characteristics of participants. A total of 16788 MRI images were obtained from 452 patients. The poor, average, good and excellent groups included 100, 100, 100, 152 patients and 3762, 3708, 3730, 5588 images, respectively. Except BMI, the other indicators were statistically significant.

**Table 1:** Patient characteristics^a^.

Variables	Poor group	Average group	Good group	Excellent group	p-value	test
No.	100	100	100	153	/	
AGE,y	66.75 ± 10.11	62.6 ± 9.96	59.18 ± 10.04	51.90 ± 14.10	P < 0.0001	Chi-squared
Gender, famale/male	67/33	73/27	59/41	72/81	P < 0.0001	One-way ANOVA
No. of images	3762	3708	3730	5588	/	
Height, cm	162.58 ± 6.43	163.16 ± 5.70	163.82 ± 5.63	165.49 ± 7.70	P < 0.0001	Kruskal-Wallis
Weight, Kg	59.16 ± 6.86	58.48 ± 5.88	61.38 ± 5.08	61.39 ± 7.50	0.0006	Kruskal-Wallis
BMI	22.35 ± 2.16	22.08 ± 2.05	22.84 ± 1.79	22.36 ± 1.91	0.0568	One-way ANOVA
Pain time, m	222.26 ± 247.86	139.45 ± 253.73	67.59 ± 130.32	30.23 ± 85.38	P < 0.0001	Kruskal-Wallis
C-reactive protein (CRP), mg/l	10.72 ± 24.18	6.10 ± 20.01	4.68 ± 9.90	2.20 ± 5.38	0.0005	Kruskal-Wallis
White blood cell (WBC), 10^9/L	6.51 ± 2.00	6.17 ± 2.02	5.48 ± 1.61	6.16 ± 2.29	0.004	Kruskal-Wallis
Neutrophil percentage, %	63.92 ± 10.78	59.67 ± 12.04	62.28 ± 9.09	61.29 ± 11.12	0.0437	Kruskal-Wallis
Erythrocyte sedimentation rate (ESR), mm/h	20.33 ± 21.99	18.78 ± 18.33	15.09 ± 14.74	13.22 ± 12.83	0.004	Kruskal-Wallis
Blood glucose, mmol/l	5.86 ± 2.27	5.57 ± 1.40	5.51 ± 1.29	5.48 ± 1.24	0.2673	Kruskal-Wallis
Extension angle, °	1.15 ± 3.47	0.39 ± 2.15	0.65 ± 3.60	0.62 ± 2.82	0.3394	Kruskal-Wallis
Flexion angle, °	108.1 ± 14.90	118 ± 11.14	117.24 ± 14.87	120.82 ± 12.89	P < 0.0001	Kruskal-Wallis
Visual Analogue Scale (VAS)	4.67 ± 0.60	4.21 ± 0.16	4.18 ± 0.54	4.41 ± 0.58	P < 0.0001	Chi-squared

^a^Values are presented as mean ± SD unless otherwise indicated.

All MRI images from a single patient were exclusively assigned to one cohort (training/validation/test) to maintain patient-level data integrity.

**Fig 3:**
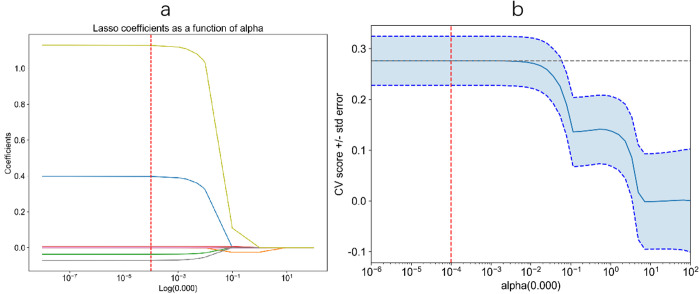
Panel a. The most valuable features were screened out by tuning λ using LASSO regression with 10-fold cross-validation via minimum binomial deviation. The dotted vertical line represents the optimal log (λ) value. Panel b. Cross validation score from the LASSO regression cross-validation procedure was plotted against λ.

**Fig 4:**
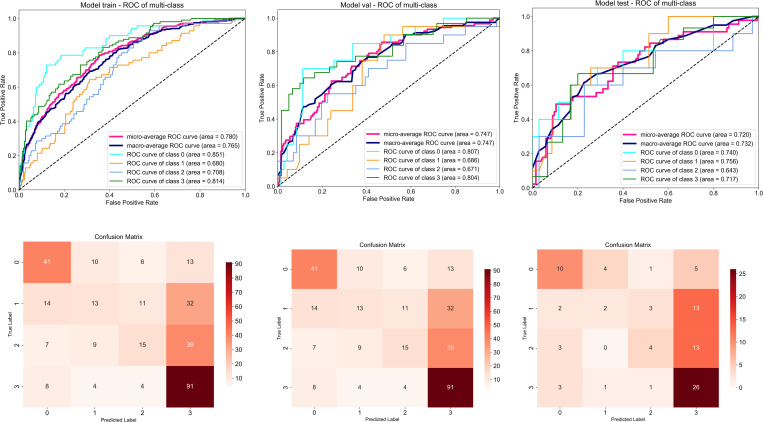
Performance and confusion matrix of the models built from imaging data only through deep learning.

Table 2 lists the model performance of deep learning under the training set, test set and validation set. The accuracy of the test set is slightly lower than that of the validation set, with ACC of 0.75 and AUC of 0.71. Fig 3 shows the variation of coefficient and cross validation (CV) score in lasso regression with the hyper parameter α. Thus, the best α value is obtained, which is about 10^−4^ to 10^−5^. Fig 4 show the deep learning ROC curve and confusion matrix. The areas of micro average ROC curve and macro average ROC curve are 0.720 and 0.732 respectively.

**Table 2:** Model Performance and Accuracy of 3-Fold Cross-validation Based on DL Models.

	Training cohort				External validation cohort				External validation cohort			
	fold1	fold2	fold3	Mean±SD	fold1	fold2	fold3	Mean±SD	fold1	fold2	fold3	Mean±SD
ACC	0.845	0.678	0.536	0.69 ± 0.15	0.846	0.604	0.725	0.73 ± 0.12	0.778	0.733	0.733	0.75 ± 0.03
AUC	0.851	0.68	0.708	0.75 ± 0.09	0.807	0.686	0.671	0.73 ± 0.07	0.74	0.756	0.643	0.71 ± 0.06
Sensitivity	0.729	0.643	0.929	0.76 ± 0.15	0.7	0.9	0.55	0.72 ± 0.18	0.6	0.7	0.6	0.63 ± 0.06
Specificity	0.879	0.688	0.425	0.66 ± 0.23	0.887	0.521	0.775	0.73 ± 0.19	0.829	0.743	0.771	0.78 ± 0.04
NPV	0.919	0.872	0.955	0.92 ± 0.04	0.913	0.949	0.859	0.91 ± 0.05	0.879	0.897	0.871	0.88 ± 0.01
PPV	0.63	0.369	0.314	0.44 ± 0.17	0.636	0.346	0.407	0.46 ± 0.15	0.5	0.438	0.429	0.46 ± 0.04
F1	0.675	0.469	0.469	0.54 ± 0.12	0.667	0.5	0.468	0.55 ± 0.11	0.545	0.538	0.5	0.53 ± 0.02

ACC: Accuracy; AUC: Area Under ROC Curve; NPV: Negative Predictive Value; PPV: Positive Predictive Value; F1: F1-Score

After combining the features obtained by deep learning with the known clinical features, different machine learning algorithms are used to obtain the joint model. Among them, light GBM is the best in the training set, test set and validation set, and their ROCs are more than 0.85. Due to the large amount of data, it will be shown in the form of charts below.

**Fig 5:**
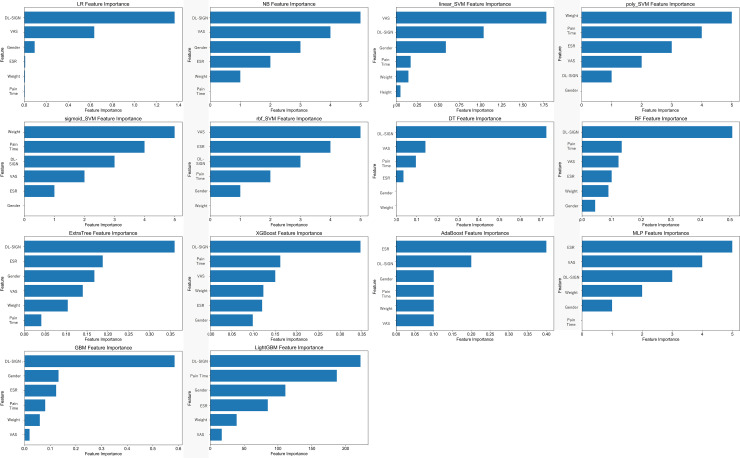
The importance of features obtained from each machine learning model.

Fig 5 shows the SHAP visualization of features obtained from each machine learning model, most of which are dl-sign.

S1, S2 and S3 Fig show the ROC curves and AUC values during the validation of each model under the training set, test set and validation set.

S4, S5 and S6 Fig show the confusion matrix of each model under the training set, test set and validation set, reflecting the specificity and sensitivity of each model.

**Fig 6:**
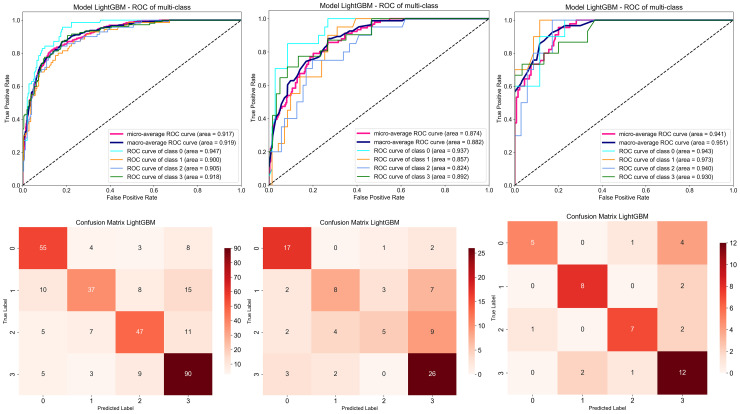
Performance and confusion matrix of the optimal model (Light GBM) constructed by combining clinical features with deep learning features.

The light GBM classifier has the highest accuracy in predicting the effect of arthroscopic meniscus plasty for degenerative meniscus tear.(Fig 6)

To facilitate reproducibility and academic exchange, we have made our model code publicly available. You can access the repository at: https://github.com/410312774/PixelMedAI/tree/main/note2-%E6%B7%B1%E5%BA%A6%E5%AD%A6%E4%B9%A0%E5%88%86%E7%B1%BB/MobHyNet. And the minimal anonymized dataset are shown in S1 Data.

## Discussion

The most significant finding of the current study is that combining deep learning image features with clinical features and using machine learning to predict the prognosis of degenerative meniscus tears undergoing APM achieves high accuracy, enabling preoperative diagnosis and treatment strategies to be provided to clinicians and patients. Among numerous classifiers, the Light GBM classifier algorithm demonstrates the highest accuracy. The average accuracy on the training set reached 0.94. Due to the potential over fitting and uneven data distribution, the average accuracy of the test set and the validation set are not consistent, but both have reached a high accuracy, which are 0.93 and 0.92 respectively.

Previous studies have shown that due to the numerous influencing factors, particularly the inability to accurately assess the condition of cartilage in patients with degenerative meniscus tears before surgery, the outcomes of arthroscopic surgery for degenerative meniscus tears are uncertain [36]. As a result, it is often difficult to predict postoperative outcomes preoperatively, leading to situations where surgeons must inform patients and evaluate future results based on the condition of cartilage damage observed during the procedure. This poses certain challenges for both clinicians and patients before surgery. Our current study establishes a postoperative prediction model for degenerative meniscus tears, which utilizes deep learning to analyze MRI data to extract features, and then combines these features with clinical data for machine learning, allowing us to accurately predict patients’ postoperative outcomes preoperatively.

The Light GBM model showed improved ROC-AUC, but PPV remains low, It is mainly due to the small sample size of the poor prognosis category (‘poor ‘/’average’) in the data (only 200 cases in total, accounting for 44.2%). Category imbalance leads to conservative prediction tendency of the model for minority categories, and false positive (FP) increases, thus reducing PPV. This is consistent with the difficulty of minority class recognition reflected by the low F1 score (0.53). Although PPV is low, the core value of the model lies in high AUC (0.92) and stratification ability: 1. it can accurately distinguish the prognosis grade (such as’ excellent ‘vs’ poor’). 2. high NPV (0.88) suggests that the model has strong reliability in excluding adverse prognosis, which helps to avoid unnecessary surgery. PPV only affects the ‘high-risk’ interpretation. In future research, we will alleviate it by adjusting the weight of loss function, threshold optimization and other methods.

**Fig 7:**
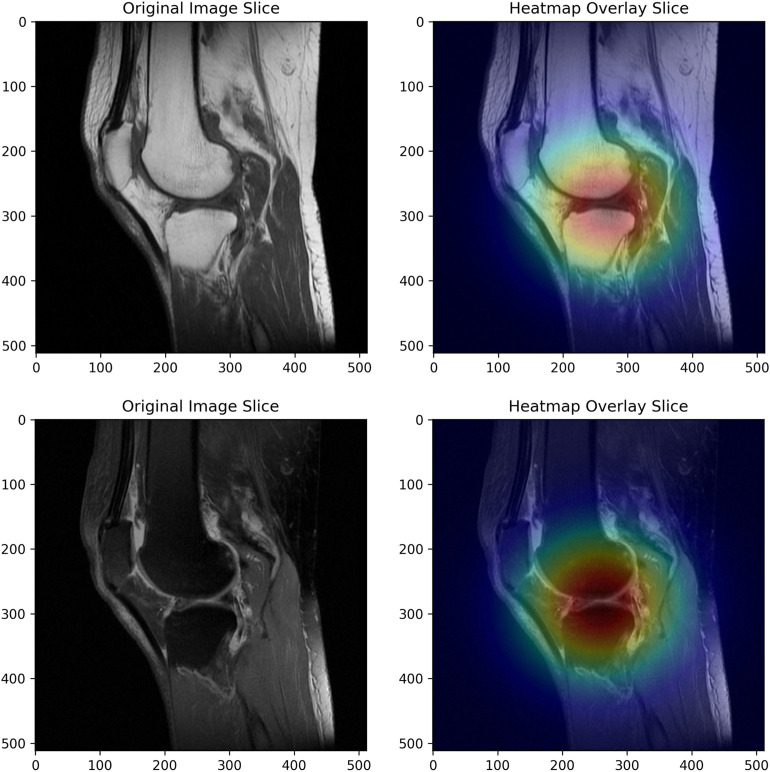
The learned feature maps of MobileNetV2 for MRI images.

Deep learning is like a black box. We can obtain feature values from deep learning, but we cannot know how the feature values are obtained or the meaning of each feature. In order to prove the reliability of this deep learning model, we use Grad-CAM visualization to visually analyze the deep learning model. As shown in Fig 7, the highlight area of the model is located in the meniscus and cartilage area of the knee joint, which also proves the reliability of the features obtained by the deep learning model.

In the model training process of this study, a series of key hyperparameters and training strategies were explicitly set to ensure the reproducibility and scientific rigor of the model’s performance. The Adam optimizer was employed for model training with an initial learning rate of 1 × 10 ⁻ ^3^. When the validation set performance showed no improvement for 5 consecutive epochs, the learning rate was automatically reduced to 0.1 times its original value. The batch size was set to 32, the maximum number of training epochs was 200, and an early stopping strategy was adopted: training was terminated early if the validation set macro AUC did not improve for 30 consecutive epochs.

The loss function used was weighted categorical cross-entropy. To address the class imbalance problem, weighted oversampling was applied to balance the class distribution of the training samples. Simultaneously, during training, z-score standardization and random rotations (−30° to + 30°) were applied to the three-dimensional image data to enhance robustness. Neural network parameters were initialized using a normal distribution, with the weights of conv3d and linear fully connected layers initialized according to the inverse square root of the number of input units. BatchNorm3d weights were initialized to 1 and biases to 0. During the training and validation process, the training loss, validation loss, and macro AUC were accurately recorded for each epoch, and corresponding curves were plotted in the final report to monitor overfitting and convergence trends. All experiments were conducted on an NVIDIA RTX 4060Ti graphics card with 24GB of memory.

Analyzing the provided training and validation curves, the training loss decreases rapidly in the early stages, with a significant reduction occurring within approximately 50 epochs. This indicates that the model can quickly learn and fit the training data. As training continues, the training loss further decreases slowly and eventually stabilizes, converging to a low level, reflecting the model’s strong fitting ability on the training set.(S7 Fig)

The training set AUC shows a steady upward trend as training progresses and eventually converges between 0.75 and 0.78, suggesting that the model can distinguish between different classes reasonably well. However, the validation set AUC also increases steadily, ultimately stabilizing between 0.70 and 0.75. Both AUC curves exhibit good convergence. Overall, the performance of the current training-validation curves indicates that the model has learned some features.(S8 Fig)

**Fig 8:**
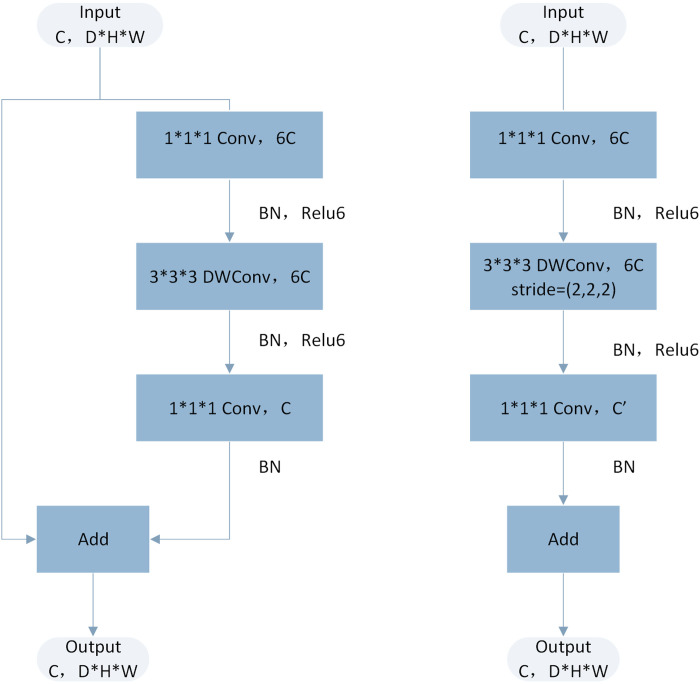
Architecture of mobilenetv2 model after 3D adaptation.

We adapted the mobilenetv2 model in 3D (Fig 8), which is a convolutional neural network designed for efficient computation. The model uses the concepts of “reverse residual” and “linear bottleneck” to achieve a balance between accuracy and model size. This adaptation replaces 2D operations with their 3D counterparts, such as “nn”. Conv3d, “nn. Batchnorm3d” and “f.avg_pool3d” modules convert 2D images into 3D volumes. The model first defines utility functions, which create convolution layers through batch normalization and relu6 activation, and are customized for 3D data. This architecture uses a new component called “reverse residual” module. The module includes an optional expansion stage, followed by the separable convolution in the depth direction, and finally the linear convolution. If the expansion is adopted, the nonlinearity is not applied to the output characteristics. The use of residual connections is conditional and is only applied when the input and output sizes are the same, which greatly alleviates the problem of vanishing gradient in deeper networks.

Although we have targeted optimized the deep learning network, the performance is still unsatisfactory. The reason is that there are many factors that affect the postoperative effect of degenerative meniscus tear. Therefore, the postoperative effect of degenerative meniscus tear can’t be accurately evaluated only from imaging or clinical information, which is the same as the research results of other scholars in the past.

Multimodal deep learning networks strongly complement the single modal drawback by providing additional information about magnetic resonance imaging and clinical changes. Therefore, we establish a deep learning machine learning model under multimodal to improve the performance of the model. After machine learning combined with deep learning features, we found that the joint model has significantly higher accuracy and better performance.

Given that this study focuses on middle-aged and elderly patients with degenerative meniscal tears, who generally have lower functional demands for their knees compared to younger individuals, we did not use the commonly utilized evaluation forms such as Lysholm Knee Scoring Scale and International Knee Documentation Committee (IKDC) Subjective Knee Evaluation Form [37,38]. Instead, we placed greater emphasis on the patients’ subjective satisfaction with their daily life, as this aligns with the goals of such surgeries. Therefore, we used a Likert scale as the outcome variable in this study.

One of the goals of artificial intelligence is to assist humans in image interpretation. Several studies have found that DL outperforms experts in medical image interpretation [39–43]. For meniscus tears, most studies mainly establish diagnostic models [44–48]. Different CNN network structure models have been established using deep learning, which can diagnose the degree of meniscus tears on MRI and compare it with human experts. These algorithms have been proven to be very useful for inexperienced or even experienced surgeons to identify before surgery. Although humans can diagnose meniscus injuries in this situation, DL systems may be useful for situations where humans cannot recognize any wireless logical relationships or patterns. Computer vision sometimes outperforms humans in recognizing more complex and subtle patterns. Therefore, DL based algorithms are expected to become more useful in interpreting various images.

To the best of our knowledge, this is the first study integrating the clinical and radiography information of degenerative meniscus tear patients to predict the effect of postoperative after arthroscopy. Although the MDLR model has shown impressive accuracy (AUC > 0.92), its clinical application faces significant challenges:. First, the imaging studies used in our research only extracted sagittal plane TI and T2 sequences and did not extract coronal and horizontal plane sequences. the exclusive reliance on sagittal-plane MRI sequences represents a notable methodological constraint. While sagittal images effectively capture anterior-posterior meniscal pathology, critical 3D spatial relationships observable in coronal and axial planes remain unexamined. Coronal sequences are essential for evaluating meniscal root integrity, extrusion magnitude, and compartment-specific cartilage wear patterns—features strongly correlated with post-APM outcomes. Concurrently, axial planes provide optimal visualization of meniscocapsular separation and popliteal hiatus abnormalities. The omission of these orthogonal planes (coronal/axial) may compromise the model’s ability to extract comprehensive biomechanically relevant features, potentially diminishing its predictive accuracy for complex degenerative tears. Furthermore, as this study did not include data on patient’s lower limb alignment prior to surgery, the accuracy of the resulting model is not perfect. Third, this study used a retrospective design and this study did not use an external validation set, the absence of external validation poses significant concerns regarding clinical deployability. Our single-center design inherently incorporates institution-specific biases in MRI protocols, surgical techniques, and demographic factors. Without multi-center validation across diverse populations and imaging environments, the model’s generalizability remains unproven. Future studies should prioritize prospective validation in geographically distributed cohorts using standardized MRI protocols incorporating all three anatomical planes to establish true world applicability. Fourthly, prognostic stratification depends on patient satisfaction (Likert scale) rather than objective functional scores (such as Lysholm/IDKD). This may introduce bias and limit comparability with other studies. Moreover, imbalanced training data can tilt the model performance towards most classes. We hope to address the mentioned shortcomings to establish a more accurate and comprehensive predictive model in future research.

## Conclusion

We have developed a model based on MDLR. Apart from the imaging features, we believe that the time of pain is an important prognostic factor for patients with degenerative meniscus tear undergoing arthroscopic partial meniscectomy.

This model is outstanding in the stratification of prognosis effectiveness of patients with degenerative meniscus tear after knee arthroscopic partial meniscectomy, and may help orthopedic surgeons preoperatively identify patients likely to have poor outcomes after surgery, supporting more informed patient counseling and potentially reducing unnecessary surgeries. It also highlights the importance of integrating imaging and clinical data for individualized treatment planning. But need for further prospective validation before clinical application.

## Supporting information

S1 Fig Performance of various machine learning models with deep learning features under training set. (TIF)

S2 Fig Performance of various machine learning models with deep learning features under verification set. (TIF)

S3 Fig Performance of various machine learning models with deep learning features under test set. (TIF)

S4 Fig Confusion matrix of various machine learning models with deep learning features under training set. (TIF)

S5 Fig Confusion matrix of various machine learning models with deep learning features under verification set. (TIF)

S6 Fig Confusion matrix of various machine learning models with deep learning features under test set. (TIF)

S7 Fig Training and validation loss of the model. (TIF)

S8 Fig Training and validation macro AUC of the model. (TIF)

S1 File MobileNetV2 architecture and formula derivations. (DOCX)

S1 Data Minimal anonymized dataset. (RAR)

## Abbreviations

MDLR: multimodal deep learning radiology
DLR: deep learning radiology
DL: deep learning
APM: arthroscopic partial meniscectomy
MRI: magnetic resonance imaging
ROC: receiver operating characteristic
ACC: Accuracy
AUC: Area Under ROC Curve
NPV: Negative Predictive Value
PPV: Positive Predictive Value
FI: F1-Score
GBM: Gradient Boosting Machine
OA: Osteoarthritis
PFP: patellofemoral pain
RCTs: randomized controlled trials
BMI: body mass index
WBC: white blood cell
ESR: erythrocyte sedimentation rate
LASSO: Least Absolute Shrinkage and Selection Operator
AUC: area under curve
IKDC: International Knee Documentation Committee
