# Supplementary figures and images for: A multimodal deep learning radiomics model for predicting degenerative meniscus tear after arthroscopy
Source: PLoS One. 2025 Aug 13;20(8):e0328299. doi: 10.1371/journal.pone.0328299 (PMC12349716; doi:10.1371/journal.pone.0328299)

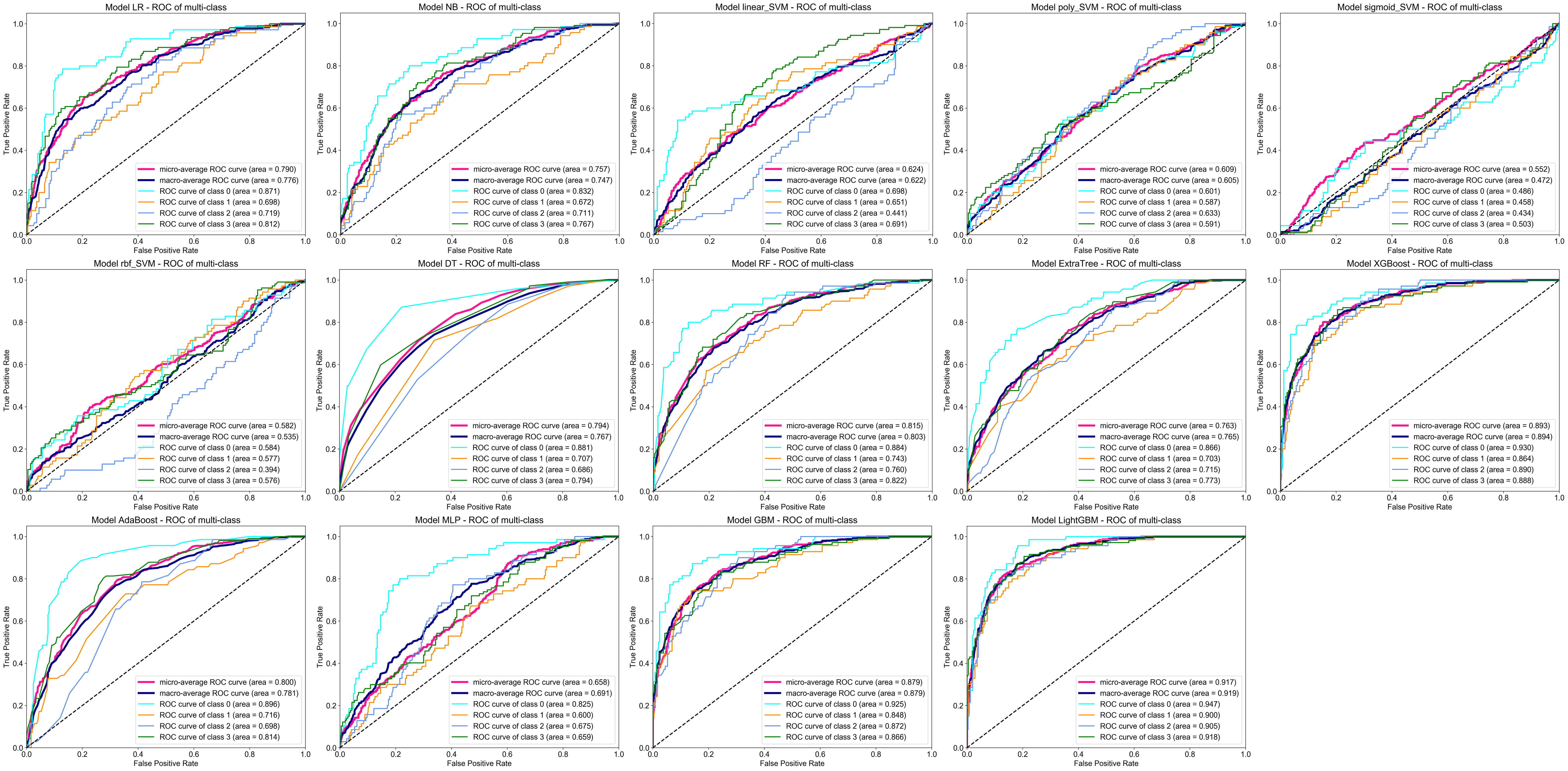

Supplement: S1 Fig — (TIF) [file pone.0328299.s001.tif]

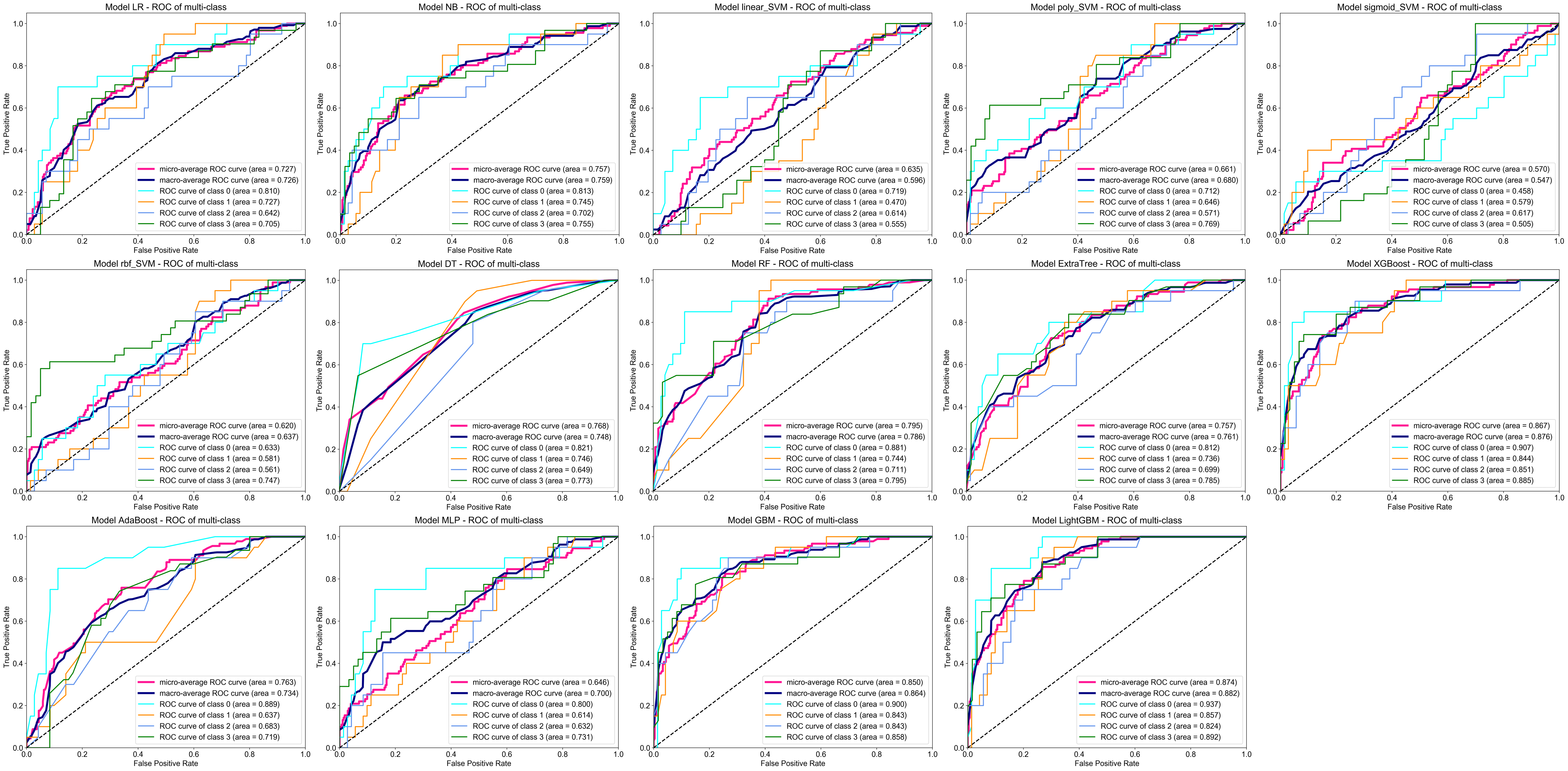

Supplement: S2 Fig — (TIF) [file pone.0328299.s002.tif]

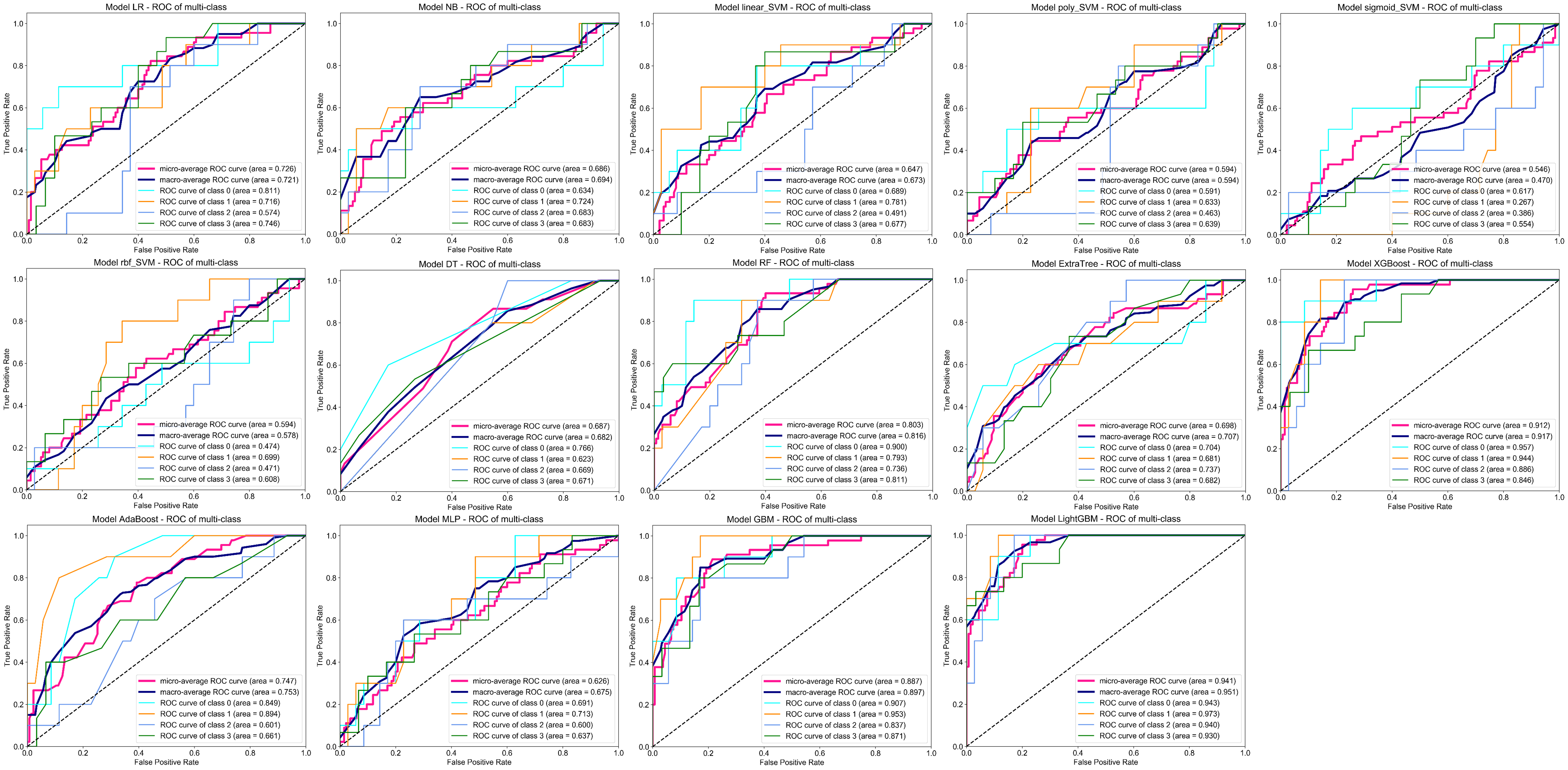

Supplement: S3 Fig — (TIF) [file pone.0328299.s003.tif]

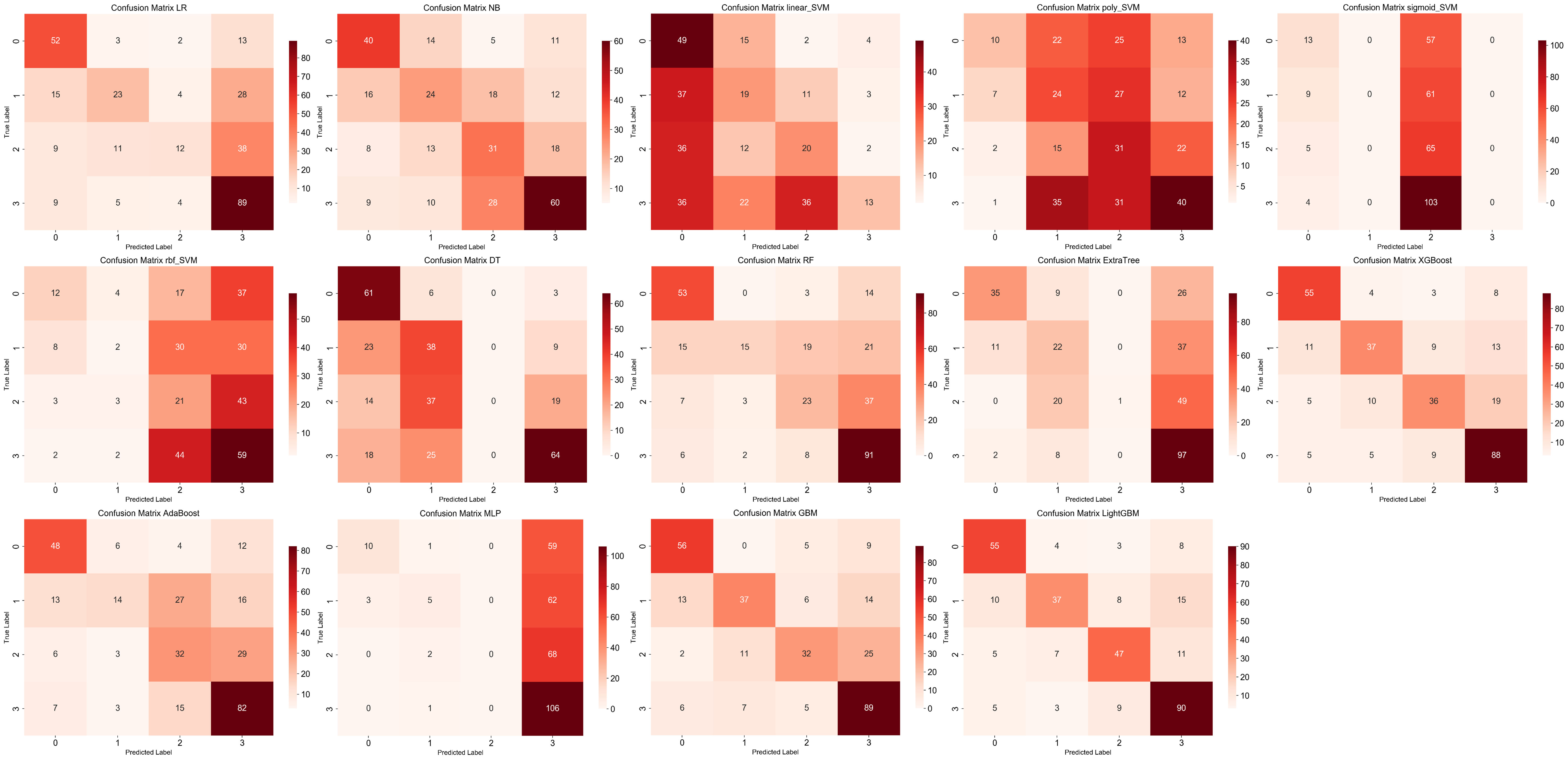

Supplement: S4 Fig — (TIF) [file pone.0328299.s004.tif]

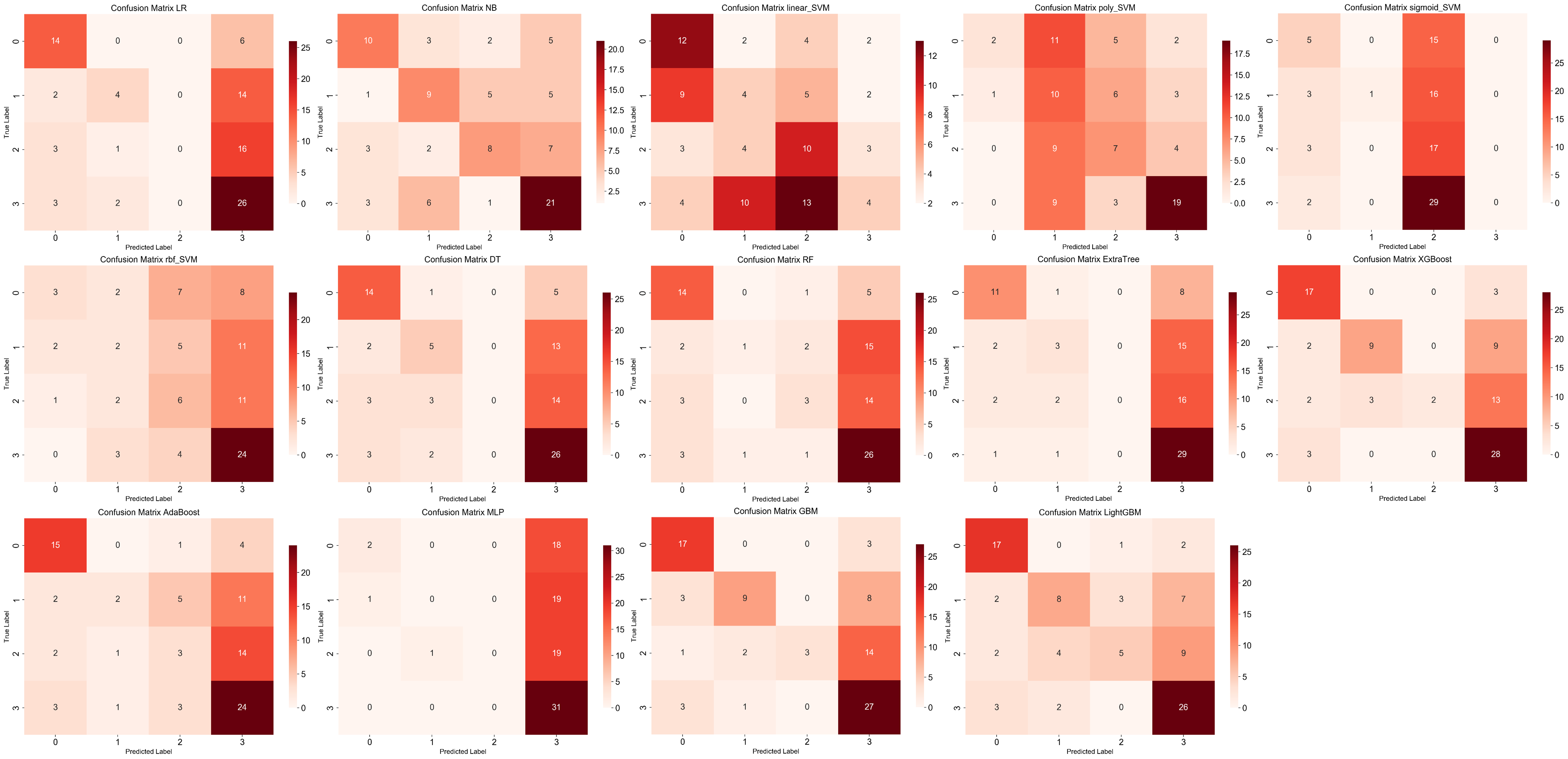

Supplement: S5 Fig — (TIF) [file pone.0328299.s005.tif]

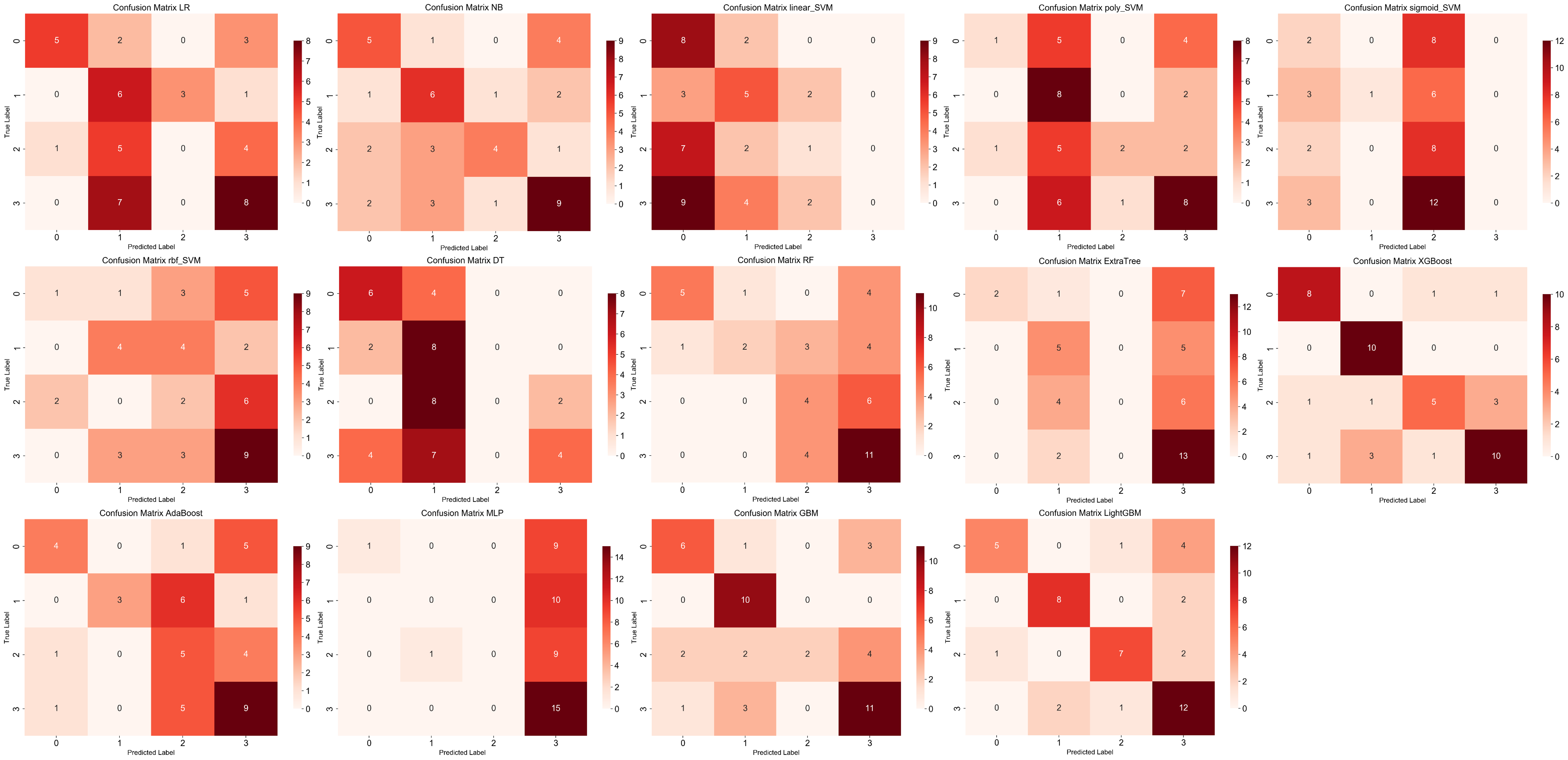

Supplement: S6 Fig — (TIF) [file pone.0328299.s006.tif]

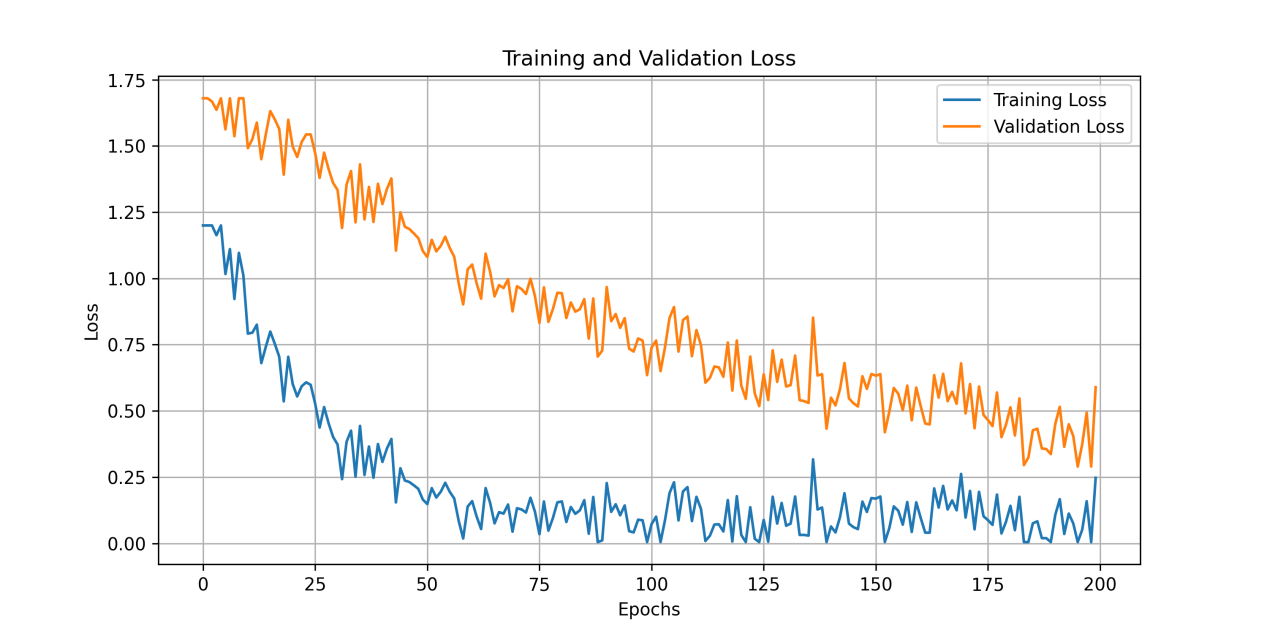

Supplement: S7 Fig — (TIF) [file pone.0328299.s007.tif]

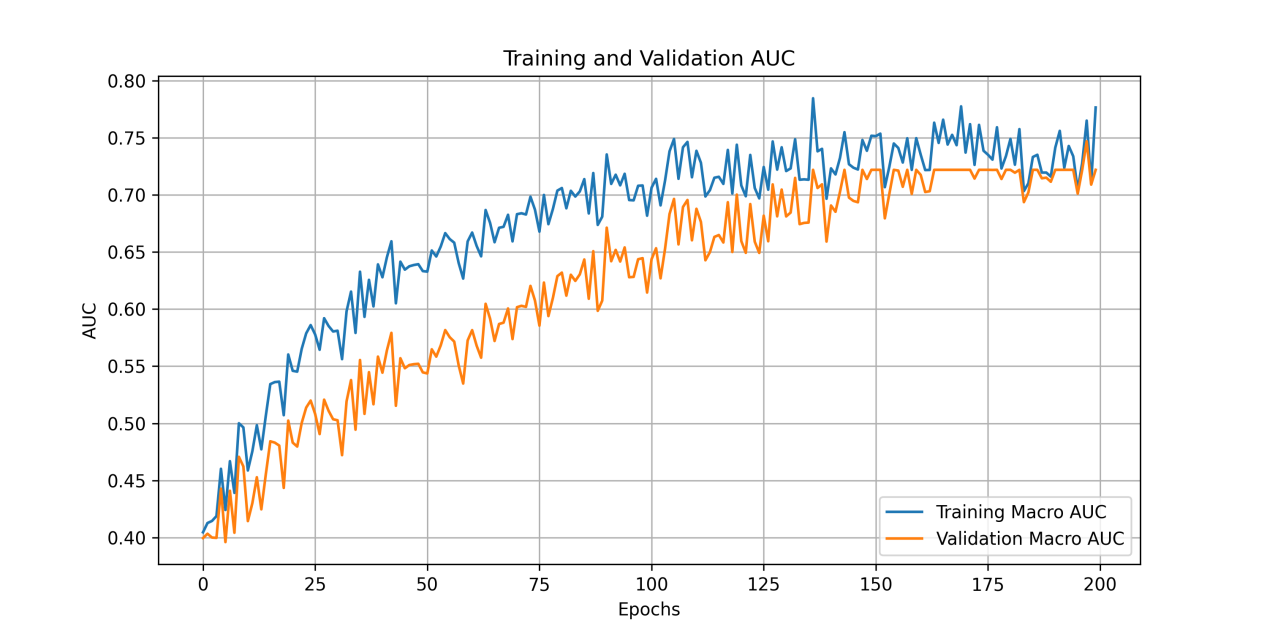

Supplement: S8 Fig — (TIF) [file pone.0328299.s008.tif]
