# Supplementary material for: A multimodal deep learning radiomics model for predicting degenerative meniscus tear after arthroscopy
Source: PLoS One. 2025 Aug 13;20(8):e0328299. doi: 10.1371/journal.pone.0328299 (PMC12349716; doi:10.1371/journal.pone.0328299)
Supplement: S1 File — (DOCX) [file pone.0328299.s009.docx]

The core of mobilenetv2 architecture is composed of several sequential "reverse residual" modules, which have a global mode and reduce the spatial dimension while expanding the number of characteristic channels. The network starts with the standard convolution layer, increases the number of input channels, and then a series of reverse residual blocks specified by a predefined sequence. This sequence is carefully designed to gradually filter and extract input data by changing the expansion factor, output channel, number of repetitions and step size through continuously decreasing spatial resolution. After traversing these layers, the model will perform the final 1x1x1 convolution to adjust the size of the feature map, and then perform the global average aggregation operation on three spatial dimensions to reduce the final feature map to one dimension of each feature channel.

The generated vectors are processed by the discard mechanism and input into a fully connected layer, which maps the processed features to the required number of output classes. The specific technology used to deal with the initialization of model parameters is adopted, and the method that helps to accelerate convergence and improve the efficiency of initial training is adopted. Conv3d layer uses a normal distribution scaled by the square root of the number of input units, while batchnorm3d weight and deviation are initialized to 1 and 0 respectively, and the weight of linear layer is also initialized to a small normal distribution.

The probability of each category output from the hybrid deep learning model mobhy net is weighted to obtain DL sign. The specific formula is as follows:

$$DL-sign=\sum_{i=0}^{n-1} v_{i}\cdot p_{i}$$

Including:

-vi is the numerical value of class i, usually a monotonically increasing sequence. pi is the probability of category i.
